# Long-Term Follow-Up of Accelerated Transepithelial Corneal Crosslinking for Post-LASIK Ectasia: A Pilot Prospective Observational Study

**DOI:** 10.3389/fbioe.2021.809262

**Published:** 2021-12-22

**Authors:** Mi Tian, Xiaoyu Zhang, Weijun Jian, Ling Sun, Yang Shen, Xingtao Zhou

**Affiliations:** ^1^ Eye Institute and Department of Ophthalmology, Eye and ENT Hospital, Fudan University, Shanghai, China; ^2^ NHC Key Laboratory of Myopia, Fudan University, Shanghai, China; ^3^ Key Laboratory of Myopia, Chinese Academy of Medical Sciences, Shanghai, China; ^4^ Shanghai Research Center of Ophthalmology and Optometry, Shanghai, China; ^5^ Shanghai Engineering Research Center of Laser and Autostereoscopic 3D for Vision Care (20DZ2255000), Shanghai, China

**Keywords:** corneal crosslinking, keratectasia, laser-assisted *in situ* keratomileusis (LASIK), safety, efficacy

## Abstract

**Background:** Keratectasia after corneal refractive surgery is a rare but serious postoperative complication, and reports on accelerated transepithelial corneal crosslinking (ATE-CXL)-based treatment of patients with post-laser-assisted *in situ* keratomileusis (LASIK) ectasia are limited. Therefore, this study evaluated the long-term efficacy and safety of ATE-CXL for progressive post-LASIK ectasia.

**Methods:** This prospective observational study was conducted at the Eye and ENT Hospital, Fudan University, Shanghai, China, and 25 eyes from 25 patients with post-LASIK ectasia undergoing ATE-CXL were examined. Clinical examinations were conducted preoperatively and postoperatively to assess parameters such as manifest refraction, corrected distance visual acuity (CDVA), endothelial cell density; keratometry, corneal thickness, posterior elevation and topometric indices were measured using Pentacam; sectoral pachymetry and epithelial thickness were evaluated using optical coherence tomography. A paired t-test, Wilcoxon rank-sum test, Kruskal-Wallis test, and repeated measures analysis of variance were used for statistical analysis.

**Results:** Participants were examined for an average of 46 months. No severe complications occurred during or after ATE-CXL. CDVA improved from 0.25 ± 0.31 preoperatively to 0.15 ± 0.17 postoperatively (*p* = 0.011). Maximum keratometry decreased from 55.20 ± 8.33 D to 54.40 ± 7.98 D, with no statistical significance (*p* = 0.074), and the central corneal thickness increased from 414.92 ± 40.96 μm to 420.28 ± 44.78 μm (*p* = 0.047) at the final follow-up. Posterior elevation, pachymetry, and epithelial thickness remained stable (*p* > 0.05) throughout the follow-up. No significant differences were noted in topometric indices, except the central keratoconus index, which decreased significantly (*p* < 0.001) at the final follow-up.

**Conclusion:** Improvements in CDVA and stabilization in corneal keratometry and posterior elevation after ATE-CXL were noted at the 46-months follow-up, demonstrating that ATE-CXL is a safe and effective treatment for progressive post-LASIK ectasia.

**Clinical Trial Registration:** http://www.chictr.org.cn/showproj.aspx?proj=13701, identifier ChiCTR-OIC-16008181

## Introduction

Keratectasia after corneal refractive surgery is a rare but serious postoperative complication. Since the first reported case of post-laser-assisted *in situ* keratomileusis (LASIK) ectasia by Seiler et al. (1998) in 1998, an increasing number of such cases has been reported. Although the precise incidence of post-LASIK ectasia remains unclear, it has been estimated to be between 0.04 and 0.6% (Binder, 2007; Chen et al., 2008). Post-LASIK ectasia can cause severe and irreversible visual impairment in patients, with progressive thinning of the cornea, progressive steepening of corneal curvature, and significant increases in myopia and astigmatism. The most common risk factors related to post-LASIK ectasia are low residual stromal bed thickness, abnormal preoperative corneal topography, thin preoperative corneal thickness, and high refractive correction (Randleman et al., 2008; Spadea et al., 2012).

There has been an increase in the number of studies exploring the application of corneal crosslinking (CXL) for patients with post-LASIK ectasia (Kymionis et al., 2009a; Salgado et al., 2011; Sueke et al., 2011; Alhayek and Lu, 2015). CXL can increase the strength and biomechanical stability of the cornea through the interaction of riboflavin and ultraviolet (UV) radiation. Previous studies (Salgado et al., 2011; Alhayek and Lu, 2015) have reported that conventional CXL (C-CXL) is an effective technique for halting keratectasia progression and has a good safety profile. Accelerated transepithelial CXL (ATE-CXL) (Shen et al., 2016; Zhang et al., 2016; Tian et al., 2018; Tian et al., 2020; Zhang et al., 2020) is an advanced CXL technique that helps maintain the integrity of the corneal epithelial layer, with a higher UV irradiation intensity (45 mW/cm^2^) and shorter irradiation duration (5 min and 20 s) than C-CXL (3 mW/cm^2^, 30 min).

To the best of our knowledge, our research team (Shen et al., 2016; Zhang et al., 2016; Tian et al., 2018; Tian et al., 2020; Zhang et al., 2020) was the first to report that ATE-CXL is safe and effective for treating adult and pediatric keratoconus. However, ATE-CXL-based treatment of patients with post-LASIK ectasia has rarely been reported in the literature. Our ophthalmology department received a referral for a larger number of patients diagnosed with post-LASIK ectasia on Chinese mainland. Therefore, this study aimed to evaluate the long-term outcomes of ATE-CXL in the treatment of patients with post-LASIK ectasia.

## Materials and Methods

### Subjects

This study adhered to the tenets of the Declaration of Helsinki and was approved by the Ethics Committee of the Eye and ENT Hospital of Fudan University (Project ID: ky 2012-017). Written informed consent was obtained from all the subjects after they were informed of the nature and possible consequences of the procedure.

The study prospectively included referral patients with progressive post-LASIK ectasia treated with ATE-CXL at the Eye and ENT Hospital of Fudan University in Shanghai, China. Evidence of progressive ectasia included an increase in maximum keratometry (K_max_) or astigmatism >1 D in the last year, excessive posterior elevation on topography mapping, or thinning in corneal thickness (Gomes et al., 2015). The exclusion criteria were: 1) history of ocular disease, 2) previous ocular surgeries (except LASIK), or 3) pregnancy or lactation during the study. Patients were instructed to discontinue wearing soft contact lenses and rigid gas-permeable lenses for at least 2 and 4 weeks, respectively, before commencing the study.

### Ophthalmologic Examinations

Preoperative and postoperative examinations were performed in all the patients as follows: slit-lamp biomicroscope examination, manifest refraction, test for corrected distance visual acuity (CDVA), and endothelial cell density (ECD). K_max_, flattest meridian keratometry (K1), steepest meridian keratometry (K2), corneal astigmatism, central corneal thickness (CCT), apex thickness (AT), and thinnest corneal thickness (TCT) were measured using Pentacam (Oculus Optikgeräte, Wetzlar, Germany). At each follow-up, the same best-fit values were used to calculate corneal posterior elevation data, including posterior central elevation (PCE) and posterior mean elevation (PME) across pre- and postoperative examinations. Pentacam was also used to evaluate topography indices, including the index of surface variance (ISV), index of vertical asymmetry (IVA), index of height asymmetry (IHA), index of height decentration (IHD), keratoconus index (KI), central KI (CKI), and minimum radius of curvature (R_min_).

Sectoral pachymetry and epithelial thickness were measured using spectral-domain optical coherence tomography (RTVue-100; Optovue, Fremont, CA, United States). The measurement area included the central cornea (central area of 2 mm diameter) and paracentral cornea (central annuli with a diameter ranging from 2 to 5 mm). Patients were followed up at 1, 6, 12 months, and four-years postoperatively. All examinations were performed by the same technician.

### Surgical Procedures

All surgeries were performed by the same experienced surgeon (Zhou). The ATE-CXL treatment was performed in an outpatient clinic using a previously described procedure (Shen et al., 2016; Zhang et al., 2016; Tian et al., 2018; Tian et al., 2020; Zhang et al., 2020). The corneal epithelium was left intact, and corneal soaking with riboflavin was performed using Paracel (containing 0.25% riboflavin-5-phosphate, hydroxypropyl methylcellulose, sodium edetate, trometamol, benzalkonium chloride, and NaCl) for 4 min, and VibeX Xtra (containing 0.25% riboflavin-5-phosphate and NaCl) for 6 min, successively. A UV-A light system (Avedro’s KXL System, MA, United States) was used to apply UV radiation (intensity of 45 mW/cm^2^) with 1-s pulsed illumination for a total duration of 5 min and 20 s, delivering a surface dose of 7.2 J/cm^2^. A bandage contact lens was applied at the end of the procedure. Postoperative medications included levofloxacin (four times daily for 3 days), 0.1% fluorometholone (seven times daily initially, gradually reduced over 2 weeks), and artificial tears (four times daily for 1 month).

### Statistical Analysis

Normality was verified using the Kolmogorov-Smirnov Z test. Comparisons between preoperative and postoperative outcomes were made using a paired t-test, Wilcoxon rank-sum test, Kruskal-Wallis test, and repeated measures analysis of variance. Statistical analysis was performed using SPSS version 23.0 (IBM Corp., Armonk, NY, United States); statistical significance was set at *p* < 0.05.

## Results

In this study, we enrolled 25 eyes from 25 patients (19 males and 6 females) who underwent ATE-CXL, with a mean age of 28.16 ± 4.84 (range, 21–38) years. Participants were followed up for an average of 46 (range, 40–54) months. All surgeries were completed successfully, and no serious complications were reported during or after ATE-CXL. The operated corneas exhibited mild edema on the first postoperative day. The bandage contact lens was removed on postoperative days 1–5 after evaluating the degree of epithelialization.

### Visual Acuity

The visual acuity outcomes are presented in Table 1. CDVA (logarithm of the minimum angle of resolution) improved from 0.25 ± 0.31 preoperatively to 0.15 ± 0.17 at 46-months postoperatively (*p* = 0.011). By the last visit, 11 eyes (44%) showed improvement in one or more Snellen lines, with a maximum increase of three lines; 11 eyes (44%) remained stable; two eyes lost one line; and one eye lost two lines.

**TABLE 1:** Refraction outcomes and endothelial cell density.

	Preoperative		Last visit		*p* Value
—	Mean ± SD	Range	Mean ± SD	Range	—
Sphere (D)	−5.92 ± 5.24	(−16.00, 1.00)	−6.38 ± 5.33	(−17.25, 0.75)	0.893
Cylinder (D)	−2.74 ± 2.12	(−8.75, −0.75)	−2.68 ± 1.97	(−5.75, 0)	0.666
Spherical Equivalent (D)	−7.29 ± 5.40	(−16.88, −1.25)	−7.72 ± 5.52	(−18.88, −0.75)	0.954
CDVA (logMAR)	0.25 ± 0.31	(0, 0.70)	0.15 ± 0.17	(0, 0.40)	0.011*
ECD (cell/mm^2^)	3,096.22 ± 288.61	(2,761, 3,561)	2,930.11 ± 249.06	(2,598, 3,254)	0.089

D, diopters; CDVA, corrected distance visual acuity; logMAR, logarithm of the minimal angle of resolution; ECD, endothelial cell density; *, a significant difference compared with preoperative.

### Manifest Refraction and ECD

The spherical equivalent was −7.29 ± 5.40 D preoperatively and −7.72 ± 5.52 D at the last follow-up (*p* = 0.954). Differences between the spherical and cylindrical degrees before and after ATE-CXL were not significant (*p* > 0.05; Table 1). No significant difference was observed in ECD at the last visit than that at the baseline (*p* = 0.089; Table 1).

### Keratometry Value and Corneal Astigmatism

**FIGURE 1:**
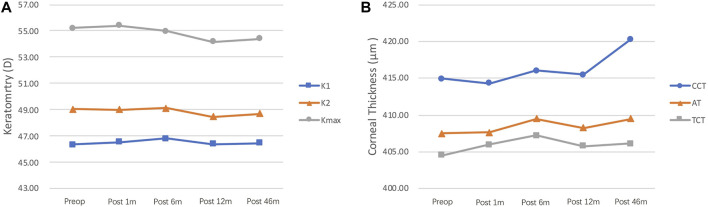
**(A)** K_max_, K1, and K2 values at different follow-up time points. No significant changes were observed in K_max_, K1, and K2 during the 46-months follow-up (*p* > 0.05). K_max_, maximum keratometry; K1, steepest meridian keratometry; K2, flattest meridian keratometry. **(B)** CCT, AT, and TCT values at different follow-up time points. The CCT value improved from 414.92 ± 40.96 μm preoperatively to 420.28 ± 44.78 μm at 46-months postoperatively (*p* = 0.047). No significant changes were observed in AT and TCT during the 46-months follow-up period (*p* > 0.05). CCT, central corneal thickness; AT, apex thickness; TCT, thinnest corneal thickness.

**FIGURE 2:**
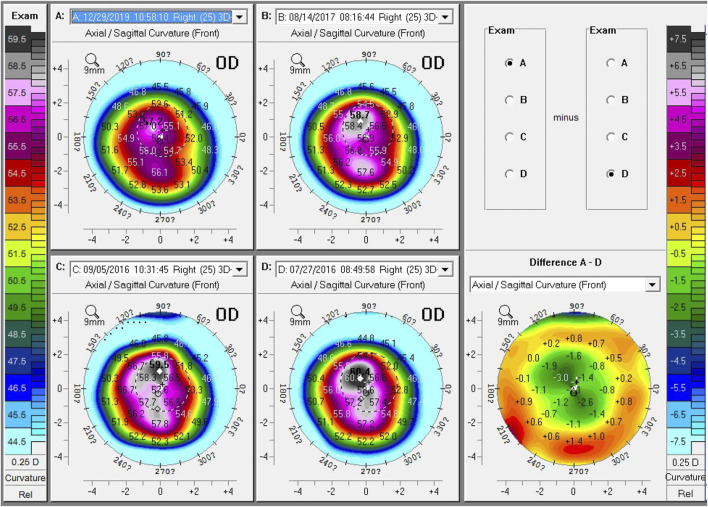
Topographic map changes of a typical case during follow-up. Comparison of preoperative and 41-months postoperative topographic maps, showing a 3.2 D decrease in K_max_, a 0.8 D decrease in K1, a 2.4 D decrease in K2, and a 1.5 D decrease in astigmatism. **(A)** postoperative 46-months; **(B)** postoperative 12-months; **(C)** postoperative 1-month; **(D)** preoperative; Difference **(A–D)**, comparison of the preoperative and 41-months postoperative front curvature.

Changes in K1, K2, and K_max_ during the 46-months follow-up are shown in Figure 1A. The K_max_ value was 55.20 ± 8.33 D before ATE-CXL, and 55.40 ± 8.44, 54.98 ± 7.87, 54.19 ± 8.03, and 54.40 ± 7.98 D at 1, 6, 12, and 46 months postoperatively, respectively. There were no significant differences in K_max_, K1, K2, and corneal astigmatism before or after ATE-CXL during the 46-months follow-up period (*p* > 0.05). The topographic map changes in a typical case are shown in Figure 2.

### Epithelial and Pachymetry Thickness

Corneal thickness, including the CCT, AT, and TCT changes at different time points, is shown in Figure 1B. The CCT value was 414.92 ± 40.96 μm before ATE-CXL, and 414.32 ± 36.85, 416.00 ± 42.03, 415.50 ± 41.89, and 420.28 ± 44.78 μm at 1, 6, 12, and 46 months postoperatively, respectively. The CCT significantly increased between the preoperative and last follow-up values (*p* = 0.047). Preoperative AT and TCT were 407.52 ± 42.43 μm and 404.56 ± 42.58 μm, respectively, and postoperative 46-months follow-up thicknesses were 409.52 ± 48.20 μm and 406.16 ± 47.51 μm, respectively, with no significant change (*p* > 0.05).

The mean thicknesses of the epithelial and pachymetry sectors before and after treatment are listed in Table 2. The pachymetry thickness of the central 2-mm sector increased from 413.48 ± 37.44 μm preoperatively, to 416.26 ± 38.33 μm postoperatively (*p* = 0.094), and the average epithelial and pachymetry thickness of neither sector showed a significant change at the last visit when compared with those at the baseline.

**TABLE 2:** Sectoral epithelial and pachymetry thicknesses (μm) (Mean ± SD).

	Sectoral epithelial thicknesses			Sectoral pachymetry thicknesses		
—	Preoperative	Last visit	*p* Value	Preoperative	Last visit	*p* Value
Min	40.91 ± 7.67	41.35 ± 7.57	0.660	393.26 ± 42.39	395.91 ± 42.80	0.134
Central 2 mm	50.39 ± 6.79	51.70 ± 7.00	0.328	413.48 ± 37.44	416.26 ± 38.33	0.094
S (2–5 mm)	58.91 ± 5.65	57.09 ± 6.51	0.090	493.91 ± 27.92	495.61 ± 30.77	0.482
SN (2–5 mm)	58.74 ± 6.22	57.65 ± 6.09	0.252	489.39 ± 25.00	490.04 ± 27.43	0.810
N (2–5 mm)	56.35 ± 7.57	55.96 ± 5.46	0.756	477.57 ± 26.24	477.83 ± 27.98	0.933
IN (2–5 mm)	53.35 ± 7.27	53.13 ± 5.51	0.857	473.87 ± 29.43	473.57 ± 31.88	0.934
I (2–5 mm)	51.35 ± 7.04	51.30 ± 5.15	0.958	474.39 ± 27.21	476.17 ± 30.36	0.646
IT (2–5 mm)	52.17 ± 6.73	50.48 ± 6.33	0.100	472.09 ± 29.59	474.74 ± 31.35	0.417
T (2–5 mm)	55.13 ± 6.18	53.04 ± 5.97	0.055	476.57 ± 32.82	478.52 ± 30.24	0.567
ST (2–5 mm)	56.78 ± 8.45	54.22 ± 6.97	0.419	487.09 ± 31.85	490.30 ± 29.18	0.225

S, superior; N, nasal; I, inferior; T, temporal.

### Posterior Elevation and Topography Indices

The mean PCE and PME values were 55.04 ± 42.22 μm and -18.72 ± 14.57 μm before ATE-CXL, and 57.76 ± 43.62 μm and −18.85 ± 13.37 μm at the last visit. There was no significant change in posterior elevation at each follow-up point (*p* > 0.05).

The preoperative and postoperative 46-months outcomes of the topography indices are shown in Table 3. The CKI value was significantly decreased from 1.08 ± 0.07 at the baseline to 1.07 ± 0.07 at the last follow-up (*p* < 0.001). The R_min_ value increased from 6.24 ± 0.91 to 6.32 ± 0.97; however, the difference was not significant (*p* = 0.068). There were no significant changes in the other evaluated topographic indices at 46 months postoperatively compared with the preoperative values (*p* > 0.05).

**TABLE 3:** Topography indices outcomes.

	Preoperative		Last visit		*p* Value
—	Mean ± SD	Range	Mean ± SD	Range	—
ISV	88.16 ± 41.92	(28, 171)	84.32 ± 41.55	(23, 167)	0.103
IVA	24.88 ± 19.78	(3.0, 81.3)	26.85 ± 20.45	(2.4, 70.5)	0.647
IHA	0.85 ± 0.51	(0.27, 1.94)	0.84 ± 0.48	(0.25, 1.67)	0.657
IHD	0.12 ± 0.08	(0.03, 0.26)	0.11 ± 0.07	(0.03, 0.27)	0.389
KI	1.21 ± 0.16	(0.97, 1.52)	1.21 ± 0.16	(0.97, 1.56)	0.873
CKI	1.08 ± 0.07	(0.97, 1.24)	1.07 ± 0.07	(0.96, 1.21)	<0.001*
R_min_	6.24 ± 0.91	(4.59, 7.81)	6.32 ± 0.87	(4.77, 7.69)	0.068

ISV, index of surface variance; IVA, index of vertical asymmetry; IHA, index of height asymmetry; IHD, index of height decentration; KI, keratoconus index; CKI, central keratoconus index; R_min_, minimum radius of curvature; *, a significant difference compared with preoperative.

## Discussion

As a surgical treatment used to increase corneal strength and stabilize the ectatic cornea, CXL has been widely adopted for the clinical treatment of keratoconus and post-LASIK ectasia. Kohlhaas et al. (2005) were the first to report the use of C-CXL in the treatment of post-LASIK ectasia. To the best of our knowledge, this study is the first to evaluate the long-term benefits of ATE-CXL in treating post-LASIK ectasia.

The patients included in the study exhibited progressive keratectasia after LASIK, with an increase of at least 1D in maximal keratometry or central corneal astigmatism within one year of the procedure. During the 46-months follow-up period after ATE-CXL, the K_max_ value decreased from 55.20 ± 8.33 D to 54.40 ± 7.98 D. No significant changes in K1, K2, corneal astigmatism, and K_max_ were observed before and after ATE-CXL during the 46-months follow-up, suggesting that ATE-CXL was effective in these cases and could halt the progression of keratectasia. Among the previous studies regarding the use of C-CXL for the treatment of progressive post-LASIK ectasia, Yildirim et al. (2014) observed that in 20 eyes, K_max_ decreased from 46.0 ± 4.4 D to 45.6 ± 3.8 D in the 42-months follow-up period, and Hafezi et al. (2007) found that in 10 eyes, K_max_ decreased from 57.4 D to 56.3 D in the two-year follow-up. Our study findings are similar to the results of these two studies, suggesting that ATE-CXL has the same effectiveness as C-CXL in the treatment of post-LASIK ectasia at long-term follow-up.

Several published studies (Kobashi et al., 2018; Stulting et al., 2018) have reported that transepithelial CXL (T-CXL) provided more rapid visual recovery in ectatic eyes than C-CXL and was superior to C-CXL by exhibiting a better CDVA at the 1-year follow-up. In our study, 46-months postoperative results showed a significantly favorable outcome in CDVA, with a mean improvement of 0.1 logMAR and a maximum increase of three Snellen lines, which indicated that ATE-CXL improved the visual acuity of ectatic patients in the long term. This might be due to the absence of corneal haze after ATE-CXL, confirmed by the lesser extent of keratocyte apoptosis and inflammation. In contrast, only a few studies have reported significant improvement in CDVA after performing C-CXL for post-LASIK ectasia (Richoz et al., 2013; Yildirim et al., 2014).

Changes in corneal thickness after CXL should be documented well since patients with post-LASIK ectasia already have a relatively thin cornea. Thinning of the cornea owing to C-CXL has been observed at the commencement of treatment and may continue up to 3 months postoperatively (Hafezi et al., 2007; Kymionis et al., 2009b); continued reduction of CCT has even been reported to occur for a duration of 3–6 years postoperatively (Kanellopoulos and Asimellis, 2014; Poli et al., 2015). The corneal thickness after ATE-CXL for keratoconus has been reported to recover and reach the baseline by 1 month (Zhang et al., 2016). In the present study, the CCT, AT and TCT values remained stable at 1, 6, 12, and 46 months postoperatively, and at the same time, the epithelial and pachymetry sector thicknesses did not show a significant change at the last visit than those at baseline, suggesting that corneal thickness remained stable after ATE-CXL throughout the 46-months follow-up period.

Regarding corneal tomographic parameters, our study found that corneal posterior elevation values showed statistically insignificant differences from preoperative values at each postoperative follow-up, suggesting that ATE-CXL prevents the corneal expansion process, thereby ensuring structural stability of the ectatic cornea. Corneal posterior elevation was reliably used to evaluate the stability of the corneal structure (Zhao et al., 2016; Zhao et al., 2017). Zhang et al. (2020) reported stable PCE values throughout the 48-months follow-up period after ATE-CXL in progressive adult keratoconus; however, PCE values were significantly increased at the three-years follow-up compared with the baseline in our previous study of ATE-CXL for progressive pediatric keratoconus (Tian et al., 2020). Therefore, the differences in pathophysiologic features between keratoconic and ectatic corneas should be investigated further.

As for corneal asymmetry parameters, CKI increases with the severity of central keratoconus (Kanellopoulos and Asimellis, 2013; Hashemi et al., 2016). The CKI value was significantly decreased at the 46-months follow-up in this study, indicating an improvement in corneal irregularities. Richoz et al. (2013) found that CKI reduced significantly at a mean follow-up of 25 months after C-CXL for ectasia after LASIK and photorefractive keratectomy and reported a significant decrease in KI, ISV, and IVA during follow-up; however, no significant differences were found in the other topographic indices in this study. Additionly, Lang et al. (2019) compared the 12-months outcomes of C-CXL with accelerated CXL and concluded that C-CXL led to greater improvements in topographic indices compared with A-CXL in patients with progressive keratoconus.

Although the efficacy of CXL has been proven, evaluating its safety is also important due to the associated cytotoxic effect of UV radiation, as well as the nonregenerative nature of endothelial cells (Thorsrud et al., 2012). Therefore, an appropriate CXL therapeutic procedure must be selected to ensure efficacy and safety. ATE-CXL treatment preserves the epithelial layer and shortens the time of infiltration and irradiation, making the patients more comfortable, accelerating postoperative recovery, conserving corneal morphology, and reducing complications. Furthermore, this approach is more suitable for patients with a relatively thin cornea. Satisfactory clinical results of T-CXL have been reported in several published articles (Yuksel et al., 2015; Chen et al., 2016; Aixinjueluo et al., 2017). Mannschreck et al. (2019) reported a case of diffuse lamellar keratitis in a post-LASIK ectatic eye that underwent C-CXL, which was unsuccessful in stopping the disease progression; however, T-CXL successfully treated the ectasia with no reported reoccurrence. In our study, no intraoperative or postoperative complications occurred, and ECD remained stable during the four-years follow-up, indicating the long-term safety of ATE-CXL treatment in patients with post-LASIK ectasia.

In our previous study (Li et al., 2018), lenticule addition followed by ATE-CXL was performed on a patient with post-LASIK ectasia. Improved CDVA and stable corneal keratometry and elevation was observed at 30 months postoperatively; thus, lenticule addition prior to ATE-CXL was proven to be a potential treatment option for patients with keratectasia associated with a thin cornea. Wallerstein et al. (2017) and El-Khoury et al. (2018) used under-flap stromal bed T-CXL for treating early post-LASIK ectasia, which facilitated early postoperative corneal stability with rapid recovery. The above methods provide promising treatment modalities for halting and stabilizing the progression of post-LASIK ectasia.

This pilot observational study had some limitations, such as the relatively small sample size and lack of a control group undergoing standard CXL. However, our results were strengthened by a long duration of postoperative follow-up. In the future, we intend to conduct a study with a larger sample size and investigate corneal biomechanical changes involved in ATE-CXL.

In summary, treatment with ATE-CXL was both effective and safe in halting the progression of keratectasia in patients with post-LASIK ectasia. Overall, the patients benefit from improvements in CDVA and stabilization in corneal keratometry and posterior elevation after a mean follow-up of 46 months, proving that ATE-CXL is safe and suitable for patients with post-LASIK ectasia.

## Data Availability

The original contributions presented in the study are included in the article/Supplementary Material, further inquiries can be directed to the corresponding author.
